# Supplementary material for: Are carbon emissions trading and green financial instruments synergistic? -Comprehensive quantitative research based on content analysis
Source: PLoS One. 2024 Mar 7;19(3):e0298601. doi: 10.1371/journal.pone.0298601 (PMC10919601; doi:10.1371/journal.pone.0298601)
Supplement: S1 Data — (ZIP) [file pone.0298601.s001.zip › Data/地级市Z_F工作报告.zip!/╜¡╦╒/╠⌐╓▌╩╨/└┤╫╘╣1⁄2╓┌║┼í╛┬φ┐╦╩2╛▌═°í┐_2006.docx]

各位代表:

现在，我代表市人民政府向大会作工作报告，请予审议，并请市政协委员和其他列席人员提出意见。

一、2005年工作和“十五”发展回顾

过去的一年，我们在中共泰州市委领导下，认真贯彻党的十六大和十六届四中、五中全会精神，紧紧围绕力争“五年总量翻番、八年全面小康”奋斗目标，全面落实科学发展观，努力走加快发展、科学发展、和谐发展之路，扎实推进全市经济、政治、文化与社会建设，较好地完成了市二届人大三次会议确定的各项目标任务。

坚持加快发展，经济总量迈上新台阶。预计全年地区生产总值突破100亿美元，达到820亿元，人均地区生产总值突破2000美元，达到16300元，可比分别增长15%和15.1%，增速创建市以来最高水平。其中，第一产业增加值89.9亿元，增长4.4%；第二产业增加值480.1亿元，增长17.9%；第三产业增加值250亿元，增长13.8%。农业生产稳步发展。弱筋小麦、优质稻米、加工型蔬菜等优势产业不断壮大，产业化龙头企业带动作用进一步增强，农业利用“三资”22亿元。市县两级农产品检测体系初步形成，畜禽高致病性传染病、水稻病虫害等防治工作得到加强。市农业开发区启动建设。供销、粮食、气象和林业绿化工作取得新成绩。工业经济快速增长。预计规模以上工业企业实现销售收入1150亿元、利税102亿元、利润53亿元，同比分别增长30%、20%和24%。积极推进“2211”工程，规模企业进一步发挥支撑作用。实施投入亿元以上重大项目40个，工业技改投入208亿元，增长35%。新创“中国名牌”产品1个、“江苏名牌”产品18个。国家医药出口基地获批，医药高新技术产业园筹建工作启动。建筑业发展势头强劲。预计全年完成建筑业总产值386亿元，增长27.7%。服务业发展速度加快。预计全年完成社会消费品零售总额233亿元，增长16.1%。社区服务、旅游业成为新的增长点。华东五金城、戴南不锈钢交易城等20个重点项目建设进展顺利，溱湖风景区被评为国家AAAA级风景区。财税金融工作取得新成绩。财政总收入突破百亿，预计达110亿元以上，其中地方一般预算收入45亿元以上，分别增长25.8%和28.8%。年末各项存款余额836.6亿元，各项贷款余额454亿元，分别比年初增长17.2%和18.1%。

坚持科学发展，增长动力得到新提升。工贸企业改革进一步深化，交通、建筑等行业企业改制步伐加快，经营性服务类事业单位改革任务基本完成。双登集团在海外成功上市。落实促进非公经济发展的政策措施，民营经济快速发展。全市新增私营企业4000户，新增个体工商户3.68万户，新增注册资本105亿元。民营经济完成增加值385亿元，税收收入48亿元，分别增长16.3％和23.7％。开放型经济实现新跨越。预计全年协议利用外资11.2亿美元，增长41.4%；实际利用外资7.22亿美元（商务部新统计口径确认数为4.5亿美元），增长73.3%。各类开发园区成为全市利用外资主阵地，海陵工业园、高港开发区进入省级经济开发区行列。外贸出口在结构优化中实现高速增长，预计完成自营出口额13亿美元，增长36.2%。对外经济技术合作进一步扩大，预计新签对外承包劳务额1.6亿美元，完成营业额1.5亿美元。境外投资项目数和投资额均列苏中第一。泰州边检站筹建工作基本完成，海关、国检、海事等口岸查验机构为开放型经济发展作出了积极贡献。沿江开发取得新突破。泰州电厂一期工程获批并开工建设，扬子江药业城项目基本完工，新扬子造船、德桥仓储、新浦化工等一批超亿美元项目进展顺利，埃力生科技工业园、嘉里粮油等项目相继落户。沿江高等级公路靖江段一期和泰兴、高港段路基桥梁工程建成，泰州过江通道工程可行性报告完成。江阴开发区靖江园区基础设施和产业项目建设全面展开。扎实推进科技进步，成功举办了第五届科技经贸洽谈会，火炬和星火计划实施、国家级高新技术企业和新产品认定、国家火炬计划特色产业基地建设等工作均在全省名列前茅，国际专利受理、国家科技进步奖获奖实现零的突破，“国家科技进步先进市”创建成功。推动工业向园区集中、人口向城镇集中、居住向社区集中，以集中布局实现集约发展。根据不同地区和产业确定土地最低投资和产出标准，促进了投资强度和产出效益的提高。

坚持统筹发展，城乡面貌发生新变化。扎实推进十大重点工程，中心城市建设总投资达62亿元。迎春桥主体、扬子江路综合改造一期等工程竣工，垃圾卫生填埋场建成投运，坡子街改造等工程加快实施，老城区污水管网一期工程全面完成，75条后街背巷改造和10个小区环境综合整治成效明显，泰山公园、环城河风景区一期等工程相继建成，东进小区定向销售房续建等工程如期竣工。强力推进“五城同创”，“创卫”通过省级调研，“创模”通过国家技术评估，“创园”通过技术验收和考核。辖市城区建设投入普遍加大，城市形象进一步提升。镇村布局规划编制完成，省、市级示范小城镇创建工作扎实推进。城乡基础设施建设取得新成绩。宁启铁路泰州站建成运营，泰高路扩建改造工程竣工，333省道兴化南绕城段建成通车；城区河道清淤整治工程竣工，“一片九河”等城市防洪工程有序推进，农村水利建设年度任务完成；220千伏双墩变、110千伏广陵变建成投运，邮电、通信等基础设施建设进一步加快。以农村“5+1”实事为抓手，进一步改善农村生产生活条件。农村自来水受益人口新增46.8万人，农村新型合作医疗参保率提高到82.2%，草危房改造任务全面完成，农村公路新建、改造1302公里，以“一免三补”为重点的农村税费改革政策提前落实到位，农村低保惠及51659人。以强化扶持为着力点，组织实施“221工程”，促进区域共同发展，黄桥老区和里下河地区30个经济薄弱乡镇主要经济指标增速可望超过全市平均水平。

坚持和谐发展，人民生活有了新改善。预计城镇居民人均可支配收入11150元，农民人均纯收入5030元，分别增长15%和10%。重视就业再就业工作，全市城镇新增就业人员3.56万人，登记失业率为3.5%。全市新转移农村劳动力7.5万人。推进“社会保险促进年”活动，养老保险、基本医疗保险覆盖面进一步扩大。组织开展失地农民社会保障试点。高度重视维护进城务工人员的合法权益。在全省率先成立市劳动争议仲裁院。注重经济社会协调发展，推动社会全面进步。高中阶段教育普及程度明显提高，初中毕业生升学率达93.1%。城乡办学条件继续改善，农村中小学“六有”和“校校通”工程建设全面完成。学校布局调整有序推进，教育资源配置得到优化。高等教育加速发展，高教园区一期工程进展顺利。乡镇文化中心标准化建设试点工作扎实推进。城隍庙修复工程完成，学政试院修缮工程开工。市佛教协会成立。光孝寺新藏经楼落成。市人民医院新病房大楼开工。市中医院进入三级甲等中医医院行列，泰兴、靖江建成国家农村中医工作先进市。市疾病预防控制中心通过国家实验室认证。普济医院建成运营。农村集镇卫生监督覆盖率达到80%。成功承办十运会泰州赛区男子篮球比赛。全市有线电视通村率达100%，城乡综合入户率60%。农村部分计划生育家庭奖励扶助和社会保障工作全面实施，人口出生率继续稳定在较低水平。整治企业违法排污成效明显，市区全年空气质量良好以上天数占比达83.3%，辖市全部创建成国家级生态示范区。外事、侨务、对台、旅游工作和新闻、统计、史志档案、社科研究、妇女儿童、老龄、残疾人事业取得新成绩。

坚持全面发展，精神文明和民主法制建设得到新加强。深入开展保持共产党员先进性教育活动，引导政府机关工作人员学习全国先进典型张云泉，争做人民群众的贴心人。大力弘扬“三创”精神，扎实开展群众性精神文明创建活动。张郭镇、黄桥镇、春兰集团、泰州中学进入全国精神文明建设先进单位行列。泰兴市通过省级文明城市复查，姜堰市、靖江市创建成省级文明城市，兴化市成为创建省级文明城市工作先进市。加强民主法制建设。认真执行市人大及其常委会的决议，及时向市政协通报情况，自觉接受人大的法律监督、工作监督和政协的民主监督。重视人大代表建议和政协委员提案工作，全年共办理人大代表建议144件，办理政协委员提案265件。推进“法治泰州”建设。基层民主法制建设继续加强，社区自治管理、“民主法制示范村”建设不断推进。“四五”普法任务完成。“平安泰州”创建活动取得成效，“大防控”体系、“大调解”机制逐步完善。贯彻信访条例，强化信访工作，落实领导下访制度，各类矛盾纠纷和信访案件得到妥善处置。推进企业、行业诚信建设，打造“诚信泰州”。工商、质量监督、安全监督、食品药品监督等部门在整顿和规范市场秩序、保障群众生产生活安全方面发挥了积极作用。成功举办首届中国泰州“战舰与城市”论坛。市人防指挥所主体工程封顶，市人防办被表彰为“全国人防建设先进单位”。政府自身建设进一步加强。认真贯彻依法行政实施纲要，行政执法水平进一步提高。继续深化行政审批制度改革，加强行政服务中心建设。推行政府信息公开，办好市长公开电话和市长信箱。强化行政效能监察，促进工作落实。廉政建设和反腐败力度加大，纠正部门和行业不正之风成效明显。

过去一年，全市经济和社会发展中还存在不少问题。一是受政策效应递减、农资价格上涨等多种因素影响，农民持续增收难度较大；二是生产性服务业发展相对滞后，服务业在国民经济中的比重仍然偏低；三是实际利用外资和民营经济总量不大，层次有待进一步提升；四是社会不安定因素依然存在，维护稳定的压力仍然较大；五是政府自身建设还存在一些薄弱环节，行政能力和服务水平需要进一步提高，等等。我们一定高度重视这些问题，采取有效措施，努力加以解决。

各位代表，刚刚过去的2005年是实施“十五”计划的最后一年。在全市人民的共同努力下，“十五”期间全市经济社会发展取得巨大成就，既定的主要发展目标圆满实现。

过去五年，是经济实力大提升的五年。预计2005年全市地区生产总值比2000年翻一番，年均可比增长13.1%，比“九五”期间年均增速提高3.6个百分点；财政总收入增长246%，其中地方一般预算收入增长173%，年均增幅分别为28.1%和22.2%；全社会固定资产投资增长188.3%，年均增长23.6%。

过去五年，是改革开放大突破的五年。工贸企业“三置换一保障”改革在全省率先推进、率先突破、率先到位，民营经济蓬勃发展，行政审批制度改革、经营性服务类事业单位改革和以农村税费改革为主要内容的农村改革成效明显。开放型经济发展势头强劲，沿江开发持续升温，一批重大产业项目相继落户。预计“十五”期间累计实际利用外资16亿美元，为“九五”期间的3.3倍，其中2005年实际利用外资比2000年增长4.83倍，年均增长42.3%；自营出口额增长295.1%，年均增长31.6%。

过去五年，是城乡建设大推进的五年。“十五”期间中心城市建设累计完成投资220亿元，比“九五”期间增加100多亿元；建成区面积由“九五”期末的37.5平方公里扩大到50平方公里，城市人口达到46.5万人，城市功能和形象得到进一步提升。辖市城区和重点中心镇面貌大变，全市城镇化进程不断加快，预计“十五”期末城市化率达46%，比“九五”期末提高6.6个百分点。城乡基础设施建设取得重大进展，宁靖盐高速公路、新长铁路、宁启铁路泰州段建成通车，一批水利、电力和信息基础设施相继建成。泰州电厂等一批重大项目相继落户，为今后发展打下了基础。

过去五年，是社会事业大发展的五年。科技对经济增长的贡献份额进一步提高，人才工作得到加强；基础教育水平不断提高，职业教育发展加快，高等教育发展取得突破，2所本科层次的院校建成招生；各类文体设施建设投入力度加大，城乡卫生体系进一步健全，抗击“非典”取得重大胜利；妇女儿童、人口与计划生育、民政福利、社会救助等社会事业发展取得新进展。环境质量综合指数在全省名列前茅，可持续发展能力进一步增强。

过去五年，是人民生活大提高的五年。与2000年相比，城镇居民人均可支配收入增加4145元，农民人均纯收入增加1481元；城镇居民人均住房建筑面积、农村住户人均住房使用面积分别增加7.8平方米和6.7平方米；年末全市城乡居民人均储蓄余额增加5368元。城镇养老、失业、医疗三大保险基本全覆盖，城市低保和农村低保实现应保尽保。挂钩帮扶黄桥老区和里下河地区经济薄弱乡镇、促进“4050”人员就业、帮助困难家庭子女入学、救助特困家庭等工作都取得了新成效。

总之，过去五年是我市综合实力提升快速、城乡面貌变化明显、人民群众得到实惠较多的五年。这些成绩的取得，是全市人民在中共泰州市委的领导下，团结一致，开拓进取，创造性工作的结果，与市人大、市政协的监督、支持也是分不开的。在此，我代表市人民政府，向辛勤工作在全市各行各业的广大干部群众，向给予政府工作热情支持和有力监督的全体人大代表、政协委员，向各民主党派、工商联、无党派爱国人士、各人民团体和广大离退休老同志，向中央和省驻泰机构以及驻泰部队指战员、武警官兵和公安干警，向所有关心支持泰州发展的海内外朋友们表示衷心的感谢！

二、“十一五”发展的目标任务

各位代表，“十一五”期间是我市全面建成小康社会的关键阶段。认真谋划好“十一五”发展，意义重大，影响深远。2004年以来，市政府组织专门力量开展了“十一五”规划的编制工作。党的十六届五中全会以后，按照中央和省委的要求，根据市委二届十次全会通过的《中共泰州市委关于制定泰州市国民经济和社会发展第十一个五年规划的建议》，市政府制定了《泰州市国民经济和社会发展第十一个五年规划纲要（草案）》，提请这次大会审议。

“十一五”期间，我市经济和社会发展的总要求是：以邓小平理论、“三个代表”重要思想为指导，全面贯彻落实科学发展观，围绕“把泰州建设得更加美好”的总体要求，以建成全面小康社会为总目标，以加快发展为主题，以提升经济科学发展水平、城乡统筹发展水平、区域协调发展水平、社会和谐发展水平为战略重点，以增强改革推动力、开放带动力、自主创新力、资源环境承载力为战略支撑，加快新型工业化、经济国际化、城市化、农业产业化进程，构筑大交通、大产业、大开放、大联动、大城市的发展格局，努力建设经济繁荣、社会文明、生活安康、环境友好的和谐新泰州。

　　“十一五”期间，我市经济和社会发展的主要目标是：经济持续快速协调健康发展，地区生产总值年均可比增长12％，力争比“十五”期末翻一番，人均地区生产总值达到4000美元左右；经济综合竞争能力明显增强，自主创新能力显著提升，形成一批拥有自主知识产权和知名品牌、核心竞争力较强的优势企业；人民群众富裕程度和生活质量明显提高，城乡之间、区域之间、社会成员之间收入分配差距扩大的趋势逐步缓解；就业相对充分，社会保障体系基本完善，公共服务体系更加健全，基本实现教育现代化，民主法制、精神文明建设全面加强，社会治安和安全生产状况进一步改善，构建和谐社会取得明显成效；经济增长方式实现较大转变，科技进步、人力资源对经济增长的支撑作用明显增强，资源利用效率显著提高，单位生产总值能源消耗比“十五”期末下降20％左右，生态市建设取得阶段性重要成果，环境污染得到有效治理，生态和人居环境有较大改善；社会主义市场经济体制比较完善，开放型经济水平全面提升。到2010年，总体上全面建成小康社会。

贯彻上述总体要求，实现主要发展目标，必须重点在推进新型工业化、经济国际化、城市化、农业产业化上下功夫，着力构建大交通，培育大产业，推进大开放，实施大联动，建设大城市。

在工作重点上，一是推进新型工业化进程。更加注重速度和结构、质量、效益相统一，促进产业结构优化升级，努力把泰州建设成为长三角现代制造业基地；更加注重依靠科技进步和人力资源开发，完善自主创新机制，不断增强科技创新能力；更加注重经济发展与人口、资源、环境相协调，大力发展循环经济，建设资源节约型、环境友好型社会。二是推进经济国际化进程。进一步提升我市经济国际化水平，在更高层次、更广领域参与国际竞争与合作。大力实施产业链和基地化引资策略，进一步加强省级经济开发区、医药高新技术产业园、沿江重点园区、重点企业主题园区的建设和发展，加快沿江开发步伐。加快转变外贸增长方式，优化出口市场和产品结构，增强企业和产品的国际竞争力，提升对外贸易的质量和效益。实施“走出去”战略，鼓励和引导企业到境外投资、开展工程承包和对外劳务合作。三是推进城市化进程。加强城乡规划的统一管理，实现城乡规划全覆盖。立足于做大、做强、做优、做美中心城市，坚持新区建设与旧城改造并举，加快基础设施、公共服务设施和商业街区、社会事业项目的规划与建设。推进辖市城区建设，构建各具特色和优势的中等城市。对黄桥、戴南-张郭两个规模较大、发展前景较好的重点区域，加快向小城市方向培育。按照地域相近原则，采取小城镇组团方式发展城市片区，全面提升小城镇整体功能。到2010年，城市化水平达55％。四是推进农业产业化进程。坚持用现代产业的理念经营农业，用现代科技武装农业，培植一批具有区域特色和市场优势的农业主导产业。推进农业龙头企业与外资嫁接，与一流科研院所合作，与品牌流通企业联手，加快培育一批骨干型、出口型、成长型重点龙头企业。大力发展农民专业合作组织、规模种养大户、农民经纪人队伍，进一步提高农民参与市场竞争的组织化程度。

在发展指向上，一是构建大交通格局。按照“1223”现代化大交通格局（一条长江黄金水道，宁启、新长两条铁路线，江阴、泰州两座长江公路大桥，宁通、宁靖盐、江海三条高速公路），建成江（都）海（安）高速泰州段、沿江高等级公路，开工建设泰州公路过江通道和宁通高速泰州段扩容工程，规划建设宁启、新长铁路泰州区域支线，推进沿江港口建设和资源整合。到2010年，高速公路里程达260公里，港口拥有万吨级以上码头泊位37个。二是构建大产业格局。全力打造医药、机电、化工、船舶等四大产业基地，2010年四大产业基地营业收入达到2000亿元；加大十大特色产业集群培育力度，2010年十大特色产业集群营业收入达到1000亿元；重点抓好“50强”工业规模企业，力争2010年营业收入过100亿元的企业5家，过50亿元的10家，过10亿元的35家；发展大物流、大市场、大商贸，打造服务业“30强”规模企业，把泰州初步建成区域性物流中心、商贸中心和长三角新兴的休闲度假旅游基地。三是构建大联动格局。放大靖江与江阴联动开发效应，促进和形成沿江地区与苏南的整体联动、全面联动；大力推进与长三角城市在信息、规划、科技、产权、旅游、交通、人才等方面的合作，加快我市融入长三角一体化进程；推进沿江与腹地的产业、人流、资本和园区的联动开发，带动黄桥老区及里下河地区加快发展。四是构建大开放格局。坚持对外开放与对内开放并重，坚持一、二、三产业开放并举，坚持三外齐抓、三外齐上，“十一五”期间，力争引进1亿美元或10亿元人民币以上的项目30个，实际利用外资累计40亿美元，民营经济注册资本年均增长20％以上，2010年自营出口额达到30亿美元。五是构建大城市格局。坚持走布局集中、城镇集聚、土地集约的城市化发展道路，加快形成“一大三中两小多片”的城镇体系框架。积极创造条件，实施市区行政区划调整，将姜堰纳入市区总体规划范围，拓展中心城市发展空间，构筑布局合理、交通便捷、功能完善、竞争力强的组团城区发展格局。

各位代表，关于今后五年的国民经济和社会发展，中央的大政方针已定，我市的宏伟蓝图已经绘出。我们坚信，经过全市人民的不懈努力，泰州必将发展得更快更好，“十一五”奋斗目标一定能够圆满实现！

三、2006年工作安排

各位代表，今年是全面贯彻落实十六届五中全会精神的重要之年，是实施“十一五”规划的起步之年，是地级泰州市组建十周年。做好今年的政府工作，具有极其重要的意义。

今年政府工作的总体要求是：深入贯彻党的十六大和十六届五中全会精神，认真落实市委二届十次全会的决策部署，以科学发展观统揽经济社会发展全局，坚持富民优先、科教优先、环保优先、节约优先，推进城乡统筹、结构调整、开放开发、环境建设、社会进步，提升三个文明建设水平，促进全市经济社会全面协调可持续发展，努力实现“十一五”发展的良好开局。

今年全市国民经济和社会发展主要调控目标初步安排为：地区生产总值增长12%，其中第一产业、第二产业、服务业增加值分别增长4%、15%和11.5%；财政收入增长18%，其中地方一般预算收入增长18％；全社会固定资产投资增长20%；社会消费品零售总额增长13%；自营出口额增长23%；实际利用外资增长30%；城镇居民人均可支配收入增长9%，农民人均纯收入增长8%；居民消费价格涨幅控制在4%以内；城镇登记失业率控制在4%以内；万元地区生产总值能耗下降4%。

今年，我们将着重做好六个方面的工作：

1、以建设社会主义新农村为方向，抓好“三农”工作。按照“生产发展、生活宽裕、乡风文明、村容整洁、管理民主”的要求，紧紧抓住促进农民增收这一中心任务，积极推进新农村建设。

大力发展现代农业。继续培大扶强农业产业化龙头企业，开展农民专业合作组织和行业协会“四有”创建活动；组织实施“三高”工程，加快建设200万亩优质水稻基地、100万亩弱筋小麦基地和“大佛指”银杏、里下河闸蟹等优势农产品产区；大力发展畜禽规模养殖，完善防疫体系；加强良种繁育推广体系、农产品标准化体系建设，培育一批具有竞争优势的农产品品牌；拓宽农业投入渠道，大力推进农业利用“三资”工作，积极探索政府与农发行之间的政策性金融合作模式；培育新型农业科技园区和农产品加工贸易区，抓好市农业开发区建设。完成林业绿化面积10万亩。完善基层农技服务体系，不断强化公益性服务职能。加强农资市场监管，维护农民合法权益。积极稳步引导土地流转，提高农业集约、规模经营水平。做好第二次全国农业普查年度工作。

积极塑造新型农民。大力实施素质教育、技能培训和创业扶助“三大工程”，积极培育有文化、懂技术、会经营的新型农民。制定农民素质教育行动计划，广泛开展星级文明户创建活动。建立健全培训网络体系，全年培训农村初高中毕业青年和农民20万人次以上。鼓励和扶持务工经商农民返乡创业，农村党员干部带头创业，种养大户和个体经营者二次创业，培育一批农民创业领头人、农民企业家。

规划建设康居村庄。按照“布局合理、设施配套、环境整洁、村容美化”的要求，坚持规划先行、因地制宜、尊重民意、注重实效，推进村庄建设和管理。科学编制村庄建设规划，引导沿江地区和中心城市城郊村庄成为城市新片区、黄桥老区和里下河地区村庄成为新社区；突出抓好“康居示范村”创建工作，逐步完善卫生、文化、商贸、环保等配套设施，建成一批道路硬化、庭院净化、村庄绿化、功能优化的新村庄示范点；扎实推进农村环境综合整治，全面开展“清洁田园、清洁水源、清洁家园”活动；探索建立村庄环境长效管理机制，启动农村社区服务和管理社会化试点。

2、以转变经济增长方式为重点，提升产业发展水平。

加快新型工业化进程。坚持以信息化带动工业化，抓好10家企业信息化示范工程，引导企业在研发、制造、营销、管理等领域应用信息技术；加快发展新材料、新能源、新医药、生物工程、电子信息等高新技术产业，积极运用高新技术和先进适用技术改造提升纺织、冶金、食品、建材等传统产业，尤其是大力推广节能、节地、节材、节水技术；鼓励和支持企业开发拥有自主知识产权的核心技术，突出抓好一批省级以上高新技术产品开发和亿元以上重大技改项目实施，确保工业技改投入占固定资产投资的比重达50%以上；扎实推进医药高新技术产业园启动区建设，支持规模企业发展主题园区，做大规模、做强实力；引导企业家加快知识更新，创新发展理念和管理体制，增强企业发展内动力。

大力发展现代服务业。积极培育现代物流、旅游休闲业，做大做强传统商贸、金融保险、社区服务业，努力提高服务业占地区生产总值的比重。规划泰州港综合物流园区，加快物流信息平台建设。积极发展连锁、代理、配送等现代营销方式，规划建设区域性商贸中心和重点市场。推进金融体制和机制创新，加快组建地方商业银行和农业合作银行，吸引国内外金融机构来泰设立分支机构。重视发展证券、信托、保险、典当等行业。整合旅游资源，强化市场促销，培育旅游品牌。继续推进环城河风景区和溱湖风景区建设。加强新型社区建设，筹建社区服务网络中心。扶持发展信息服务业。规范和发展各类中介机构。培育房地产市场，促进房地产业发展。

做大做强建筑业。发挥比较优势，做大重点市场，重点拓展北京、深圳、上海、重庆四大建筑市场；做强龙头企业，推动优质资源向江苏一建、江苏中兴、江苏正太三家特级资质企业和“十强”企业集中；做优经营结构，引导企业主攻规模特色项目；做响企业品牌，进一步扩大“建筑之乡”影响。

3、以增强经济发展动力为核心，进一步推进改革开放。

力争招商引资新突破。继续“推进大招商、主攻大项目、建设大园区”，力争在引资规模、项目层次和载体建设上取得新突破。充分发挥沿江优势、产业优势和企业优势，重点招引全球500强和国内100强等领军企业，力争新增3－5个单体超亿美元的重大项目；以特色化、外向化、规模化为方向，重点建设省级开发区，加快形成功能更加完善、特色更趋鲜明、带动更为有力的园区发展新格局。紧紧抓住世界贸易持续增长的机遇，加快转变外贸增长方式，保持外贸出口快速增长。继续实施“走出去”战略，大力拓展对外工程承包和劳务合作市场，积极发展境外加工贸易。

开创沿江开发新局面。推进项目开发、功能提升、资源整合、两岸联动和生态建设，打造沿江产业带、城镇带、风光带。加快实施泰州电厂等一批在建重大项目，开工建设埃力生科技工业园等一批已批重大项目，争取签约一批新的重大项目；继续完善沿江基础设施，推进沿江高等级公路建设，抓紧做好泰州公路过江通道开工准备，确保泰州港永安港区一期工程竣工营运，加快泰州口岸远程监控系统建设和新建码头对外开放；加快岸线资源整合，优化岸线布局结构，促进腹地产业发展；继续推进跨江联动开发，江阴开发区靖江园区基础设施和产业投入取得新突破；规划建设沿江防护林，启动建设江心洲湿地生态区，尽快实现沿江地区污水集中达标排放，打造沿江绿色工业走廊和生态景观走廊。

推动各项改革新进展。以建立现代企业制度为方向，进一步深化工贸企业改革，加快建筑企业改制步伐，基本完成外贸、交通企业改制任务。稳步推进投融资体制改革，完善企业投资项目核准和备案制度。抓好公益类事业单位改革。稳步推进乡镇机构改革，整合乡镇事业站（所）。继续深化行政审批制度改革，精简审批事项，加强市行政服务中心建设和管理。

　　促进民营经济新发展。继续弘扬“三创”精神，强化典型引导和创业辅导，进一步做好全民创业促进工作。引导民营企业加快机制创新、技术创新、管理创新，重点支持100家民营企业建设技术中心，打造知名品牌，参与“W3100”上市工程，尽快发展壮大。以放宽准入、公平竞争、公平待遇为重点，落实鼓励非公经济发展的政策意见，进一步改善民营经济发展环境。健全民营经济社会化服务体系，中小企业担保机构新增注册资本8000万元以上，建立泰州民营经济服务网。

4、以强化统筹为着力点，促进区域、人与自然协调发展。牢固确立统筹发展理念，构建城乡一体发展、区域共同发展、人与自然和谐发展的新格局。

加快城市化进程。继续突出中心城市的龙头地位，协调推进城镇建设，全年中心城市建设投资力争完成75亿元，全市城市化水平比上年提高1.5个百分点。坚持以重大工程推进带动城市功能完善、以重点区域建设带动城市面貌改善、以重要路段改造带动城市形象提升，充分发挥市、区两级共同推进中心城市建设的积极性，明确责任，完善机制，力争完成中心城市建设三年实施纲要目标任务。加快编制城市分区建设规划和控制性详细规划，优先覆盖周山河街区、旧城改造区和城乡结合部。新城区建设重点向南推进、拉开框架、拓展空间，实施东风南路、鼓楼南路、海陵南路南延及三路联接线工程，并启动开发建设相应区域，推进高港城区金港南路改造、向阳路北延二期等重点工程建设；老城区建设继续改善环境、完善功能、提高品位，实施环城河二期、青年路改造等重点工程，完成东西城河贯通、迎春路、东大门等景观改造，加快坡子街商业中心建设和东风北路两侧地域、城北地区危旧房改造，基本完成后街背巷改造，启动迎春桥西北侧街区改造。坚持建设与管理并重，不断提高城市管理水平。继续引导辖市城区创新城建理念，重视新区建设、功能完善和环境营造。引导重点中心镇完善规划，科学定位，加快建设，彰显城镇特色和个性。

完善城乡基础设施。加强城乡路网建设，建成姜八公路泰兴段，启动兴泰公路新（扩）建、盐兴邮公路兴化段改造等一批干线网化工程，新建、改造农村公路1000公里。加强城乡水利建设，继续实施中心城市新区防洪封闭工程，启动老城区东北片防洪工程，完成农村河道疏浚、圩区治理年度任务和城黄灌区五期改造工程。加强城乡电网建设，完成兴化唐子、姜堰王石等4项重点输变电工程，开工建设500千伏江都至泰兴（泰州段）线路工程。加强城乡信息基础设施建设，实施电信、移动、联通网络扩容工程，创建区域性无线电监测中心。完善市人防指挥所功能。

促进区域共同发展。从构建区域协调互动机制入手，推动黄桥老区和里下河地区经济薄弱乡镇加快发展。健全利益分配和业绩考核机制，鼓励和引导经济薄弱乡镇到重点园区和沿江地区“飞地”开发；健全合作机制，支持沿江地区和腹部地区开展多种形式的经济协作和技术、人才、劳务合作；健全扶持机制，按照公共服务均等化原则，落实扶持政策和扶持资金，强化部门挂钩帮扶，促进经济薄弱乡镇经济社会协调发展。

建设资源节约型、环境友好型社会。毫不动摇地坚持“三集中”的方针，大力发展技术、投资、劳动、效益密集的工业、农业和服务业，带动节约型经济体系建设。编制和实施循环经济建设规划，抓好循环经济示范企业、行业和工业园区试点，重点建设8个示范园区。组织修编全市第二轮土地利用总体规划，加大土地开发、复垦、整理力度，促进土地合理、集约和可持续利用。在全社会深入开展以节水、节地和节能为重点的节约活动，大力提倡绿色消费、文明消费。突出抓好中心城市环境综合整治，完成滨江工业园区污水处理厂及高港区污水管网建设，扩大老城区污水支管网覆盖面，继续加强清洁能源区、噪声达标区、烟尘控制区建设，开展打击企业违法排污和保护饮用水源地水环境专项行动，实施扩绿添景工程，年内新增绿化面积100万平方米。加快乡镇特别是重点中心镇污水处理设施建设，扎实开展农村小康社会环保行动，建成市级以上环境优美乡镇4个、生态村10个。

5、以构建和谐社会为目标，推动社会全面进步。坚持以人为本的发展导向，更加注重社会事业发展，更加注重社会公平和稳定，更加注重民主法制建设，促进社会和谐。

突出抓好就业再就业和社会保障工作。积极发展就业容量大的劳动密集型产业，开展“创业带动就业”行动，着手建立城乡统一的人力资源市场，完善“4050”人员再就业援助制度，认真落实促进就业的各项优惠政策，全年新增城镇就业人员2.5万人。完善养老保险制度，改革计算办法，着手做实个人帐户，逐步提高统筹层次。强化社会保险扩面征缴，全年新增参保人员10万人。推行劳动保障一卡通。强化劳保监察工作，维护企业职工和进城务工农民的合法权益。加大被征地农民社会保障制度推进力度，努力将城镇规划区、工业园区等用地集中区新征地农民纳入保障范围。巩固农村低保成果，完善农村新型合作医疗制度。继续做好城市贫困家庭帮困工作，逐步解决农村五保户供养标准和集中供养比例偏低的问题。进一步健全完善社会救助体系和灾害应急机制，支持社会慈善、社会捐赠、群众互助等社会扶助活动。

优先发展科技、教育事业。继续完善科技创新体系，加快市高新技术创业中心等“孵化器”建设，引导重点企业建好“一站两中心”；实施“百企三创”工程，推动百家高新技术企业创自主技术、创优质产品、创知名品牌；开展“企业院校行”活动，探索建立产学研相结合的有效机制。办好博鳌亚洲论坛国际医药产业大会和长三角城市经济协调会。加强科学普及。进一步巩固和完善“以县为主”的义务教育管理体制，不断加大教育投入。整合、放大优质教育资源，促进教育均衡协调发展。加快合格学校建设，推进教育现代化工程。重视和加强师资队伍建设，推进素质教育，减轻学生负担。完成高教园区一期工程，确保秋季新生入学。加大人才培养、引进和使用力度，改善人才创业环境，构建人才高地。

繁荣各项社会事业。实施泰州市民族民间文化保护工程，加强乡镇文化中心标准化建设。加快市文化中心建设，实施乔园修复扩建工程。继续推进有线广播电视进村入户，稳步发展数字电视。进一步完善公共卫生体系，强化农村卫生设施建设和基本医疗保健服务，大力推进城市社区卫生服务工作，加大艾滋病、结核病等重点传染病的防控力度。逐步调整优化农村水厂布局，推进区域联网供水。全面落实计划生育社会保障优惠政策，着力抓好流动人口和社区计划生育工作。深入实施全民健身计划纲要，进一步提高竞技体育水平。实施市体育中心二期工程。启动地方志续修工作。建立市域地名公共服务系统。重视民族宗教工作。积极发展新闻、气象、抗震等事业，继续做好妇女儿童、老龄、残疾人工作。

加强精神文明建设。继续抓好“五城同创”和“四市联创”，制定、实施创建全国文明城市三年规划，力争中心城市通过国家卫生城市、国家环境保护模范城市考核验收。实施市民素质教育三年行动计划，加大文明村镇创建力度。进一步加强和改进未成年人思想道德建设和大学生思想政治教育，健全学校、家庭、社会“三位一体”工作机制。继续做好国防教育、民兵预备役、兵员征集工作。

扎实推进民主法制建设。自觉接受同级人大及其常委会的监督，落实有关决议、决定，支持人民政协履行职责，主动听取各民主党派、工商联、无党派人士以及工会、共青团、妇联等群众团体的意见和建议。加强与人大代表和政协委员的联系，认真办理人大代表建议和政协委员提案。以“四民主两公开”为主要内容，推进农村基层民主法治建设。抓好社区规范化建设，创建和谐社区。贯彻落实《建设法治泰州实施纲要》，推进依法治市。启动实施“五五”普法，提高法律援助和法律服务水平。继续推进“平安泰州”建设。发挥“大调解”机制作用，提高基层调处化解矛盾的能力。进一步落实信访工作领导责任制，构建大信访格局，妥善处置群体性、突发性事件。建立社会安全和社会治安分析预警机制，完善“大防控”体系。强化安全生产监督检查，防止重特大事故发生。实施食品、药品放心工程，保障人民群众生命健康安全。

6、以提高行政水平和服务质量为主线，加强政府自身建设。按照“为民、务实、清廉”要求，进一步加强政府自身建设，不断提高施政能力和水平，更好地肩负起建设和谐美好新泰州的重任。

坚持施政为民，竭力办好实事。忠实执行党的群众路线，把为民、利民、便民作为全部工作的立足点和出发点，努力把代表最广大人民群众根本利益的要求体现在具体行动上。深入开展下访活动，了解百姓意愿，倾听百姓疾苦，化解百姓困难。坚持从群众最关心、最直接、最现实的问题入手，认真解决征地中侵害农民利益、拆迁中侵害居民利益、工程建设中拖欠克扣农民工工资以及看病贵、上学难等热点问题。积极探索为民办实事的长效机制，继续为民办好10件实事，巩固提高农村“5+1”实事办理成果。

坚持与时俱进，提高施政水平。围绕提高科学发展能力，牢固树立正确的政绩观，进一步转变发展观念、创新发展路径、破解发展难题。围绕提高社会服务能力，在继续加强经济调节和市场监管的同时，集中精力抓好社会管理和公共服务；建立健全各种应急机制和体系，提高政府保障公共安全和应急处置能力；扩大政府信息公开，加快电子政务建设，办好市长信箱、市长公开电话。围绕提高科学决策能力，健全公众参与、专家论证和政府决策相结合的科学决策机制，适时邀请市民代表参加政府常务会议。围绕提高依法行政能力，认真贯彻《全面推进依法行政建设法治政府实施方案》，落实行政执法责任制，加快建立权责明确、行为规范、监督有效、保障有力的行政执法体制。围绕提高行政执行能力，坚持把“411工程”作为年度工作的大纲、总揽全局的抓手、部署落实的重点，进一步提高组织程度，强化效能监察，加大推进力度。

坚持从严治政，树立廉洁形象。贯彻落实《建立健全惩治和预防腐败体系的实施办法》，切实加强反腐倡廉工作。推进廉政文化建设，引导公务员强化廉洁从政意识，筑牢拒腐防变的思想防线。开展反腐倡廉制度建设推进年活动，进一步完善工程建设招投标、土地使用权出让、政府采购等制度，努力从源头上预防和治理腐败。坚持行政监督、法律监督并举，组织监督、社会监督并重，强化对权力运行的制约和监督。开展行风评议，加强经济责任审计，深化纠风和专项治理工作，着力解决群众反映强烈的突出问题，树立清正廉洁的良好形象。

各位代表，新的征程已经开启，新的使命崇高神圣。让我们紧密团结在以胡锦涛同志为总书记的党中央周围，认真贯彻十六届五中全会精神，全面落实科学发展观，在中共泰州市委的领导下，紧紧依靠全市人民，与时俱进，开拓创新，团结拼搏，扎实工作，为完成全年各项目标任务，推进“五年总量翻番、八年全面小康”进程而努力奋斗！
